# Development of the antigenic recombinant tropomyosin of *Echinococcus granulosus* in a bacterial system as a vaccinal candidate against canine echinococcosis

**DOI:** 10.17843/rpmesp.2024.414.13854

**Published:** 2024-10-25

**Authors:** Janet Acosta-Benites, Luis M. Jara, Manuela Verastegui Pimentel, Pepe M. Obregón Maldonado, Faride Altamirano-Zevallos, Nicasio Valencia Mamani, Cesar M. Gavidia

**Affiliations:** 1 Faculty of Pharmacy and Biochemistry, Universidad Nacional Mayor de San Marcos, Lima, Peru. Universidad Nacional Mayor de San Marcos Faculty of Pharmacy and Biochemistry Universidad Nacional Mayor de San Marcos Lima Peru; 2 Faculty of Veterinary Medicine and Zootechnics, Universidad Peruana Cayetano Heredia, Lima, Peru. Universidad Peruana Cayetano Heredia Faculty of Veterinary Medicine and Zootechnics Universidad Peruana Cayetano Heredia Lima Peru; 3 Laboratory of Infectious Diseases Research (LIEI), Faculty of Sciences and Philosophy, Universidad Peruana Cayetano Heredia, Lima, Peru. Universidad Peruana Cayetano Heredia Laboratory of Infectious Diseases Research (LIEI) Faculty of Sciences and Philosophy Universidad Peruana Cayetano Heredia Lima Peru; 4 Faculty of Veterinary Medicine, Universidad Nacional Mayor de San Marcos, Lima, Peru Universidad Nacional Mayor de San Marcos Faculty of Veterinary Medicine Universidad Nacional Mayor de San Marcos Lima Peru; 5 Faculty of Science and Engineering, Universidad Nacional de Huancavelica, Huancavelica, Peru. Universidad Nacional de Huancavelica Faculty of Science and Engineering Universidad Nacional de Huancavelica Huancavelica Peru

**Keywords:** Recombinant DNA, echinococcosis, Echinococcus granulosus, Tropomyosin, vaccine

## Abstract

This study aimed to clone, express and produce the recombinant Echinococcus granulosus tropomyosin isoform A protein (EgTrpA) that maintains its antigenic and immunogenic properties as a potential vaccine candidate for dogs and sheep. The Echinococcus granulosus tropomyosin protein (EgTrp) gene was cloned into two vectors: Tropo/His-tag [pET28a (+)] and Tropo/GST-tag (pGEX6P-1). It was then expressed in E. coli BL21. Protein identity was determined by two-dimensional electrophoresis. Immunogenicity and antigenicity were verified by immunizing rabbits with each recombinant protein and assessed by western blot and ELISA. Two-dimensional electrophoresis identified the recombinant EgTrp protein as isoform A. The recombinant proteins showed recognition reactions on Western Blot and serum from immunized rabbits showed an increase in Tropo/His-tag IgG antibodies similar to Tropo/GST-tag. The recombinant EgTrpA protein showed antigenic and immunogenic characteristics in laboratory animals.

## INTRODUCTION

Cystic echinococcosis is a zoonotic disease caused by the larval stage of the cestode *Echinococcus granulosus* sensu lato (sl). Its adult stage parasitizes the intestine of canines (definitive host). Definitive hosts disseminate eggs through feces into the environment, while ruminants (mainly sheep) and humans are intermediate and accidental hosts that develop hydatid cysts ^1^. The most affected regions are Central and South America (Central Highlands of Peru), East Africa and Central Asia ^2^ with a prevalence of 5-10% in endemic areas ^3^.

The interruption of the transmission of the biological cycle is important for the success of control strategies. For example, the EG95 vaccine prevents the establishment of parasite oncospheres in sheep target organs ^4^. However, immunization in the definitive host is a more cost-effective alternative, as there are often fewer canids than sheep ^5^. In addition, domestic dogs represent the greatest risk for human infection due to their close relationship ^6^.

*Echinococcus* is a genetically diverse parasite and the structural and immunological characteristics of several proteins have been studied as potential vaccine candidates ^7^. For example, an experimental vaccine with the egM protein, involved in the development of mature parasites, induced a high level of protection (97%-100%) in dogs, as measured by embryogenesis, as well as by worm growth and suppression of egg development ^8^.

Tropomyosin (Trp) is a parasitic muscle protein that has several isoforms that can be produced from the same gene ^9^. Different studies have demonstrated the importance of tropomyosin in developing protective immunity due to its high antigenicity. An experimental study demonstrated that vaccination with recombinant tropomyosin and a paramyosin-like fibrillar protein (EgA31) significantly reduced the parasite load in vaccinated dogs compared to unvaccinated dogs challenged with *E. granulosus*^10^.

Currently, despite the different studies on *E. granulosus* genes encoding antigenic proteins, the development of vaccines has limitations based on few experimental studies and the genetic diversity of the parasite worldwide, since the immunomodulatory characteristics are not fully known. This study aimed to clone, express and produce a recombinant tropomyosin protein from *E. granulosus protococci* that maintains its antigenic characteristics and immunogenic properties.

**KEY MESSAGES**

Motivation for the study. Cystic echinococcosis is a neglected disease associated with contact between dogs, humans and sheep. In countries such as Peru, control programs include vaccination of sheep; however, vaccination in dogs is a late control strategy to eliminate the adult parasite or to avoid infection with eggs in the environment.

Main findings. We were able to clone and express a recombinant protein (tropomyosin) of the adult parasite in a bacterial system with immunogenic properties.

Implications. Obtaining the tropomyosin recombinant protein from *E. granulosus* allows the development of vaccine candidates in dogs and the exploration of diagnostic tests in hosts.

## THE STUDY

### Synthesis and cloning of the tropomyosin gene of *E. granulosus*

Viable *E. granulosus protococci* (sl) were isolated from lung and liver cysts of naturally infected adult sheep (animals raised in the Central Highlands of Peru) that were slaughtered in slaughterhouses in Lima during 2022. The descriptive laboratory study was performed during 2023. The commercial kit Direct-zol™ RNA Miniprep Plus (Zymo Research, USA) was used for RNA extraction and the commercial kit SuperScript™ VILO™cDNA Synthesis Kit (Ivitrogen, USA) was used for conversion to cDNA.

Forward and reverse primers were designed from tropomyosin coding sequences using NCBI Primer-BLAST software (https://www.ncbi.nlm.nih.gov/tools/primer-blast/) and Oligo Analyzer IDT (https://www.idtdna.com/calc/Analyzer/Home). In addition, the coding sequences of antigenic proteins were analyzed using the NEBcutter V2.0 program from New England BioLabs (http://nc2.neb.com/NEBcutter2/) for the location of enzymatic restriction sites.

### Expression and purification of recombinant tropomyosin proteins

Amino acid sequence characteristics (molecular weight and isoelectric point) were determined using the ProtParam program of the ExPASy server (https://web.expasy.org/protparam/). Protein expression of the recombinant plasmids was induced following previously published protocols ^11^.

Recombinant Tropo/His-tag and Tropo/GST-tag were purified by affinity chromatography. His-Select Nickel Affinity Gel (Sigma-Aldrich) was used to purify the recombinant Tropo/His-tag protein, and GLUTATHIONE SEPHAROSE® 4B (GE Healthcare) was used for the recombinant Tropo/GST-tag protein, following the manufacturer’s instructions. 

The recombinant proteins were evaluated by two-dimensional electrophoresis ^7^.

### Assessment of immunogenicity and antigenicity

Immunogenicity was verified by detection of antibodies in rabbits immunized with Tropo/His-tag and Tropo/GST-tag. Animals were immunized four times subcutaneously at 15-day intervals (2 months); two rabbits received 130 µg of Tropo/His-tag and the other two received 150 µg of Tropo/GST-tag, using complete and incomplete Freund’s adjuvant for the first and subsequent immunizations, respectively.

The presence of antibodies against the recombinant antigen was assessed by indirect ELISA ^12^. The two recombinant antigens Tropo/His-tag and Tropo/GST-tag were coated on ELISA microplates and incubated with rabbit serum samples at 0, 15, 30, 30, 45 and 60 days after immunization. The absorbance was then measured at 450 nm with a spectrophotometer (TECAN, Magellan, USA).

The antigenicity of the recombinant protein was analyzed by Western blot ^13^ at two different concentrations. Group A used ∼0.001 mg Tropo/His-tag and ∼0.1 mg Tropo/GST-tag and group B used ∼0.01 mg Tropo/His-tag and ∼1.0 mg Tropo/GST-tag. Group A strips were incubated with serum produced in rabbits immunized with the recombinant protein and group B strips were incubated with serum produced in rabbits immunized with *E. granulosus* native antigen (EgAPM) (from the Laboratory of Veterinary Epidemiology, Universidad Nacional Mayor de San Marcos, Peru).

The experimental study was evaluated and approved by the Ethics and Animal Welfare Committee of the Faculty of Veterinary Medicine, Universidad Nacional Mayor de San Marcos (N°2022-11).

## RESULTS

### Synthesis and cloning of the tropomyosin gene from *E. granulosus*

Colony PCR of recombinant Tropo/His-tag and Tropo/GST-tag plasmids, which transformed into *E. coli* DH5α, showed products with an expected molecular weight of 858 bp for the His-tag vector and 857 bp for the GST-tag vector. Sequencing determined that both Tropo/HIS-tag and Tropo GST-tag had a 99% match to the *E. granulosus* reference sequence of EgTrpA (GenBank AF011923.3).

### Expression and purification of recombinant tropomyosin proteins

**Figure 1:**
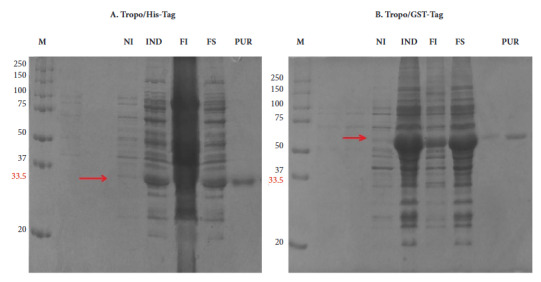
Expression of recombinant (A) Tropo/His-tag and (B) Tropo/GST-tag proteins before and after induction. (NI) *E. coli* BL21 without IPTG inducer, (IND) *E. coli* BL21 with IPTG inducer, (IF) insoluble fraction, (SF) soluble fraction, (PUR) Purified Protein (single band), (M) molecular weight marker in KDa.

Recombinant proteins expressed in *E. coli* BL21 showed an expected weight of approximately 33.5 kDa for Tropo/His-tag and 60.1 kDa for Tropo/GST-tag. The soluble fraction was used for protein purification and a single band was found after purification (Figure 1). Quantification of purified proteins by the Bradford technique had a concentration of 0.27 µg/µl for Tropo/HIS-tag and 0.16 µg/µl for Tropo/GST-tag.

**Figure 2:**
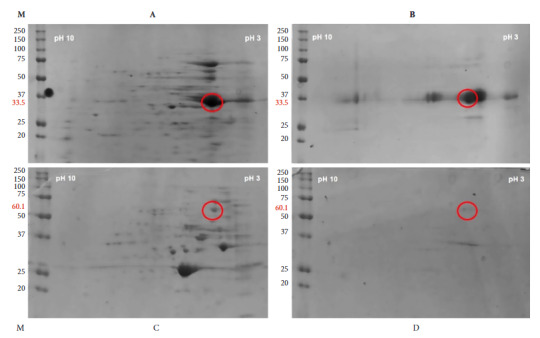
Two-dimensional electrophoresis: (A) before Tropo/HIS-tag purification, (B) after Tropo/HIS-tag purification, (C) before Tropo/GST-tag purification and (D) after Tropo/GST-tag purification

Two-dimensional electrophoresis was performed to identify the recombinant *E. granulosus* protein before and after purification (Figure 2). Sequence analysis with the ProtParam program showed an acidic pH for the EgTrpA protein (pH 4.6 for Tropo/His-tag and pH 4.8 for Tropo/GST-tag).

### Assessment of immunogenicity and antigenicity

**Figure 3:**
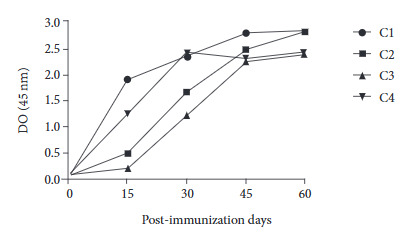
Results of indirect ELISA to detect anti-Tropo/His-tag and anti-Tropo/GST-tag IgG antibodies in rabbits immunized with recombinant tropomyosin proteins. C1 and C2 represent Tropo/His-tag immunized rabbits; C3 and C4 represent Tropo/GST-tag immunized rabbits. Day 0 represents serum collected prior to immunization.

ELISA results showed an increase in polyclonal anti-Tropo/His-tag and anti-Tropo/GST-tag antibodies obtained from rabbits immunized with the recombinant proteins (Figure 3). Two rabbits (1 and 4) showed an increase in anti-recombinant protein IgG on day 15 compared to day 0, while the other two rabbits (2 and 3) showed an increase on day 30. Immunized rabbit serum showed an increase in Tropo/His-tag IgG antibodies (OD 450 nm: 2.82) similar to Tropo/GST-tag (OD 450 nm: 2.40) on day 60.

**Figure 4:**
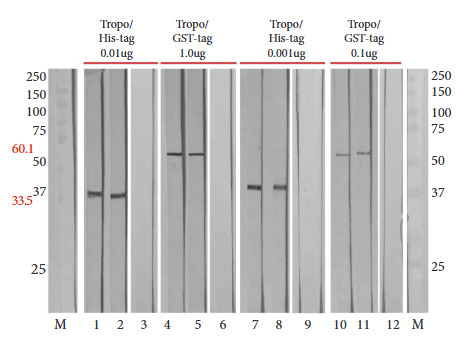
Western blot of recombinant tropomyosin recognized by serum from immunized rabbits. Lanes 1 to 6 represent serum from Tropo/His-tag and Tropo/GST-tag immunized rabbits. Lanes 7 to 12 represent serum from rabbits immunized with EgAPM (E. granulosus native antigen). La-nes 3, 6, 9 and 12 represent a set of negative serum samples from rabbits prior to immunization. Lane M represents a commercial molecular wei-ght marker.

Western blotting confirmed the antigenicity of the recombinant proteins by obtaining a single band with the expected weight of 33.5 kDa for Tropo/His-tag and 60.1 kDa for Tropo/GST-tag. The recombinant antigen reacted strongly with anti-Tropo/His-tag and anti-Tropo/GST-tag produced in the immunized rabbits (Figure 4). Tropo/His-tag reacted at a concentration of 0.001 µg, in contrast to Tropo/GST-tag, which reacted at a concentration of 0.1 µg. The recombinant antigen showed reactivity with anti-EgAPM antibodies (Figure 4).

## DISCUSSION

*E. granulosus* is a genetically diverse parasite that exhibits variable biological, biochemical, immunological and antigenic characteristics. It is important to consider the types of parasitic proteins to be used, given that the larval and adult stages produce diverse antigens that trigger different immunity or response mechanisms ^14^. In *E. granulosus*, tropomyosin is found in the larval and adult stages and is known to have high immunogenicity ^15^. In this study we selected EgTrpA because it has been reported to be the most abundant form compared with other protein isoforms ^16^.

*E. coli* was selected as the expression system because it is the most commonly used host and has often been described as efficient. More complex systems exist, and these allow the production of proteins with homologous characteristics to native proteins. However, considerable experience and practicality has been accumulated in the cultivation of *E. coli*, which has allowed a significant improvement in production without complications ^18^.

The plasmid vectors selected in this study have been widely used to produce antigenic recombinant proteins ^17^. An advantage of using commercial vectors is the presence of the His-tag and GST-Tag tail that allows purification by affinity chromatography. A disadvantage of the pET28 vector may be the high concentrations of protein generated using this system, which may result in inclusion bodies ^18^. In our study, we similarly found a high concentration of Tropo/His-tag proteins that resulted in purification difficulties. The pGEX6P-1 vector produced a low concentration and required longer production time. An advantage is the presence of the GST protein as more soluble and antigenic, which can be exploited in the development of vaccine candidates ^8^.

2-DE has contributed significantly to proteomic mapping and characterization ^13^. In our study, before purification, several protein spots were reproducibly separated by high-resolution 2-DE. After purification, samples treated with the clean-up kit did not yield a higher number of Tropo/GST-tagged spots. Even so, 2-DE allowed us to identify the absence of changes in protein expression, because, the distribution of the recombinant protein profiles was similar to those obtained from protoscolices ^13^.

The recombinant Tropo/His-tag and Tropo/GST-tag proteins were functional and immunogenic, since the immune response produced an increase of IgG in rabbits. These recombinant proteins could be used as an antigenic source for the production of polyclonal antibodies for Copro-ELISA diagnosis in dogs ^19^. In addition, anti-EgTrpA antibodies can be purified and evaluated for the development of serological tests in intermediate hosts. As a result, the production of recombinant proteins such as EgTrpA could be more affordable, feasible, and sustainable than obtaining parasite proteins from naturally infected animals.

This study has some limitations such as the fact that recombinant Tropo/GST-tag protein was not produced in sufficient quantity to immunize a large number of animals. Several repetitions of the protocol were required to obtain sufficient protein; therefore, the use of bioreactors would need to be evaluated. Another limitation was that genotyping of the parasite was not performed, although it is known that sheep cysts originate exclusively from the G1 genotype in Peru, which also commonly affects humans _^20^_. Finally, the rabbit as an animal model does not resemble the definitive host. Antibodies would not necessarily represent a correlate of protection, so egg reduction in the dog should be evaluated.

In conclusion, this study describes the successful production of EgTrpA in two vectors Trpop/His-tag and Tropo/GST-tag with homologous characteristics to native parasite proteins. The production of recombinant antigens represents a perspective application for the design and development of a vaccine against echinococcosis in dogs that requires further studies to evaluate its efficacy. This would be complementary to the preventive measures that should continue to be articulated under the One Health approach, since the biological cycle involves people, wild and domestic canids, livestock, as well as contamination of the environment with the parasite eggs.
