# Supplementary material for: Development of the antigenic recombinant tropomyosin of Echinococcus granulosus in a bacterial system as a vaccinal candidate against canine echinococcosis
Source: Rev Peru Med Exp Salud Publica. 2024 Oct 25;41(4):411–6. doi: 10.17843/rpmesp.2024.414.13854 (PMC11797582; doi:10.17843/rpmesp.2024.414.13854)
Supplement: Translation

# Translation: Desarrollo de la tropomiosina A recombinante antigénica de *Echinococcus granulosus* en un sistema bacteriano como candidato vacunal contra la equinococosis canina

**Authors:** Janet Acosta-Benites, Luis M. Jara, Manuela Verastegui Pimentel, Pepe M. Obregón Maldonado, Faride Altamirano-Zevallos, Nicasio Valencia Mamani, Cesar M. Gavidia

**Affiliations:** 1 Facultad de Farmacia y Bioquímica, Universidad Nacional Mayor de San Marcos, Lima, Peru.; 2 Facultad de Medicina Veterinaria y Zootecnia, Universidad Peruana Cayetano Heredia, Lima, Peru.; 3 Laboratorio de Investigación de Enfermedades Infecciosas (LIEI), Facultad de Ciencia y Filosofía, Universidad Peruana Cayetano Heredia, Lima, Perú.; 4 Facultad de Medicina Veterinaria, Universidad Nacional Mayor de San Marcos, Lima, Perú; 5 Facultad de Ciencias e Ingeniería, Universidad Nacional de Huancavelica, Huancavelica, Perú.

**Keywords:** ADN recombinante, equinococosis, Echinococcus granulosus, Tropomiosina, Vacuna

## Abstract

El objetivo de este estudio fue clonar, expresar y producir la proteína recombinante tropomiosina isoforma A de *Echinococcus granulosus* (EgTrpA) que mantenga sus propiedades antigénicas e inmunogénicas como posible candidata a vacuna para perros y ovejas. El gen de la proteína tropomiosina de *Echinococcus granulosus* (EgTrp), se clono en dos vectores: Tropo/His-tag [pET28a (+)] y Tropo/GST-tag (pGEX6P-1). Luego, se expresó en *E. coli* BL21. La identidad de la proteína se determinó mediante electroforesis bidimensional. La inmunogenicidad y la antigenicidad se verificó mediante la inmunización de conejos con cada proteína recombinante y se evaluó mediante Western Blot y ELISA. La electroforesis bidimensional identificó la proteína recombinante EgTrp como isoforma A. Las proteínas recombinantes mostraron reacciones de reconocimiento en el Western Blot y el suero de los conejos inmunizados mostró un aumento de los anticuerpos IgG de Tropo/His-tag similar a Tropo/GST-tag. La proteína recombinante EgTrpA demostró características antigénicas e inmunogénicas en animales de laboratorio.

## INTRODUCCIÓN

La equinococosis quística es una enfermedad zoonótica producida por la fase larvaria del céstodo *Echinococcus granulosus* sensu lato (sl). Su fase adulta parasita el intestino de los caninos (huésped definitivo). Los huéspedes definitivos diseminan los huevos a través de las heces al ambiente, mientras que los rumiantes (principalmente ovejas) y los humanos son huéspedes intermediarios y accidentales que desarrollan los quistes hidatídicos ^1^. Las regiones más afectadas son América Central y del Sur (Sierra Central del Perú), África Oriental y Asia Central ^2^ con una prevalencia del 5-10% en zonas endémicas ^3^.

La interrupción de la transmisión del ciclo biológico es importante para el éxito de las estrategias de control. Por ejemplo, la vacuna EG95 impide el establecimiento de oncosferas del parásito en los órganos diana de las ovejas ^4^. Sin embargo, la inmunización en el huésped definitivo es una alternativa más rentable, ya que a menudo hay menos cánidos que ovejas ^5^. Además, los perros domésticos presentan el mayor riesgo de infección humana debido a su estrecha relación ^6^.

Echinococcus es un parásito genéticamente diverso por lo que se han estudiado las características estructurales e inmunológicas de varias proteínas del parásito como posibles candidatas a vacunas ^7^. Por ejemplo, una vacuna experimental con la proteína egM, implicada en el desarrollo de parásitos maduros, ha inducido un nivel alto de protección (97%-100%) en perros, medido por la embriogénesis, así como por el crecimiento de los gusanos y la supresión del desarrollo de los huevos ^8^.

La tropomiosina (Trp) es una proteína muscular parasitaria que tiene varias isoformas que pueden producirse a partir del mismo gen ^9^. Diferentes estudios han demostrado la importancia de tropomiosina para desarrollar inmunidad protectora debido a su alta antigenicidad. Un estudio experimental demostró que la vacunación con tropomiosina recombinante y una proteína fibrilar similar a la paramiosina (EgA31), redujo la carga parasitaria significativamente en perros vacunados en comparación con no vacunados y retados con *E. granulosus*^10^.

Actualmente, a pesar de los diferentes estudios sobre genes de *E. granulosus* que codifican proteínas antigénicas, el desarrollo de vacunas presenta limitaciones basadas en pocos estudios únicamente experimentales y a la diversidad genética del parásito a nivel mundial, ya que no se conocen en su totalidad las características inmunomoduladoras. El objetivo de este estudio fue clonar, expresar y producir una proteína tropomiosina recombinante a partir de protoescólices de *E. granulosus* que mantenga sus características antigénicas y propiedades inmunogénicas.

**MENSAJE CLAVE**

Motivación para el estudio. La equinococosis quística es una enfermedad desatendida que se asocia al contacto entre perros, hombre y ganado ovino. En países como Perú, los programas de control incluyen la vacunación de ovinos, sin embargo, la vacunación en perros es una estrategia de control a futuro para eliminar el parásito adulto o evitar el contagio con los huevos en el ambiente.

Principales hallazgos. Se logró clonar y expresar una proteína recombinante (tropomiosina) del parásito adulto en un sistema bacteriano con propiedades inmunogénicas.

Implicaciones. La obtención de la proteína recombinante tropomiosina de *E. granulosus* permite el desarrollo de candidatos vacunales en perros y la exploración de pruebas diagnósticas en los hospederos.

## EL ESTUDIO

### Síntesis y clonación del gen de la tropomiosina de *E. granulosus*

Los protoescólices viables de *E. granulosus* (sl) se aislaron de quistes de pulmón e hígado de ovejas adultas infectadas naturalmente (animales criados en la Sierra Central del Perú) que fueron beneficiadas en mataderos de Lima durante el 2022. El estudio descriptivo de laboratorio se realizó durante el 2023. Para la extracción del ARN se utilizó el kit comercial Direct-zol™ RNA Miniprep Plus (Zymo Research, EE. UU.) y para la conversión a ADNc se usó el kit comercial SuperScript™ VILO™cDNA Synthesis Kit (Ivitrogen, EE. UU.)

Se diseñaron cebadores directos e inversos a partir de las secuencias codificantes de tropomiosina, utilizando el software NCBI Primer-BLAST (https://www.ncbi.nlm.nih.gov/tools/primer-blast/) y el Oligo Analyzer IDT (https://www.idtdna.com/calc/Analyzer/Home). Además, se analizaron las secuencias codificantes de las proteínas antigénicas utilizando el programa NEBcutter V2.0 de New England BioLabs (http://nc2.neb.com/NEBcutter2/) para la localización de los sitios de restricción enzimática.

### Expresión y purificación de las proteínas tropomiosinas recombinantes

Las características de las secuencias de aminoácidos (peso molecular y punto isoeléctrico) se determinaron utilizando el programa ProtParam del servidor ExPASy (https://web.expasy.org/protparam/). La expresión proteica de los plásmidos recombinantes se indujo siguiendo protocolos previamente publicados ^11^.

Las Tropo/His-tag y Tropo/GST-tag recombinantes se purificaron mediante cromatografía de afinidad. Para la purificación de la proteína recombinante Tropo/His-tag se utilizó el His-Select Nickel Affinity Gel (Sigma-Aldrich), y para la proteína recombinante Tropo/GST-tag se utilizó GLUTATHIONE SEPHAROSE® 4B (GE Healthcare), siguiendo las instrucciones del fabricante.

Las proteínas recombinantes se evaluaron finalmente mediante electroforesis bidimensional ^7^.

### Evaluación de la inmunogenicidad y la antigenicidad

La inmunogenicidad se verificó mediante la detección de anticuerpos en conejos inmunizados con Tropo/His-tag y Tropo/GST-tag. Los animales fueron inmunizados cuatro veces vía subcutánea con intervalos de 15 días (2 meses); dos conejos recibieron 130 µg de Tropo/His-tag y los otros dos recibieron 150 µg de Tropo/GST-tag, utilizando adyuvante de Freund completo e incompleto para la primera inmunización y para las subsiguientes, respectivamente.

La presencia de anticuerpos contra el antígeno recombinante se evaluó mediante ELISA indirecto ^12^. Los dos antígenos recombinantes Tropo/His-tag y Tropo/GST-tag se recubrieron en microplacas para ELISA y se incubaron con muestras de suero de conejo a los 0, 15, 30, 45 y 60 días posteriores a la inmunización. Luego se midió la absorbancia a 450 nm con un espectrofotómetro (TECAN, Magellan, EE. UU.).

La antigenicidad de la proteína recombinante se analizó mediante Western blot ^13^ en dos concentraciones diferentes. El grupo A utilizó ∼0,001 mg de Tropo/His-tag y ∼0,1 mg de Tropo/GST-tag y con el Grupo B ∼0,01 mg de Tropo/His-tag y ∼1,0 mg de Tropo/GST-tag. Las tiras del grupo A fueron incubadas con suero producido en conejos inmunizados con la proteína recombinante y las tiras del grupo B fueron incubadas con un suero producido en conejos inmunizados con el antígeno nativo de *E. granulosus* (EgAPM) (proveniente del Laboratorio de Epidemiología Veterinaria de la Universidad Nacional Mayor de San Marcos, Perú). 

El estudio experimental fue evaluado y aprobado por el Comité de Ética y Bienestar Animal de la Facultad de Medicina Veterinaria, Universidad Nacional Mayor de San Marcos (N°2022-11).

## RESULTADOS

### Síntesis y clonación del gen de la tropomiosina de *E. granulosus*

La PCR de colonias de los plásmidos recombinantes Tropo/His-tag y Tropo/GST-tag, transformados en *E. coli* DH5α, mostró productos con un peso molecular esperado de 858 pb para el vector His-tag y 857 pb para el vector GST-tag. La secuenciación determinó que tanto Tropo/HIS-tag como Tropo GST-tag tenían una coincidencia del 99% con la secuencia de referencia de *E. granulosus* de EgTrpA (GenBank AF011923.3). 

### Expresión y purificación de las proteínas recombinantes de tropomiosina

Las proteínas recombinantes expresadas en *E. coli* BL21 mostraron un peso esperado de aproximadamente 33,5 kDa para Tropo/His-tag y 60,1 kDa para Tropo/GST-tag. La fracción soluble se utilizó para la purificación de proteínas y se observó una única banda después de la purificación (Figura 1). La cuantificación de las proteínas purificadas mediante la técnica de Bradford tuvo una concentración de 0,27 µg/µl para Tropo/HIS-tag y 0,16 µg/µl para Tropo/GST-tag.

**Figura 1:** Expresión de proteínas recombinantes (A) Tropo/His-tag y (B) Tropo/GST-tag antes y después de la inducción. (NI) E. coli BL21 sin el inductor IPTG, (IND) *E. coli* BL21 con el inductor IPTG, (FI) fracción insoluble, (FS) fracción soluble, (PUR) Proteína Purificada (banda única), (M) el marcador de peso molecular en KDa.

Se realizó una electroforesis bidimensional para identificar la proteína recombinante de *E. granulosus* antes y después de su purificación (Figura 2). El análisis de la secuencia con el programa ProtParam indicó un pH ácido para la proteína EgTrpA (pH 4,6 para Tropo/His-tag y pH 4,8 para Tropo/GST-tag).

**Figura 2:** Electroforesis bidimensional: (A) antes de la purificación Tropo/HIS-tag, (B) después de la purificación Tropo/HIS-tag, (C) antes de la purificación Tropo/GST-tag y (D) después de la purificación Tropo/GST-tag.

### Evaluación de la inmunogenicidad y la antigenicidad

Los resultados de la prueba ELISA mostraron un aumento en los anticuerpos policlonales anti-Tropo/His-tag y anti-Tropo/GST-tag, obtenidos de conejos inmunizados con las proteínas recombinantes (Figura 3). Dos conejos (1 y 4) tuvieron un aumento de IgG anti-proteína recombinante el día 15 en comparación con el día 0, mientras que los otros dos conejos (2 y 3) tuvieron aumento el día 30. El suero de conejo inmunizado mostró un aumento de los anticuerpos IgG de Tropo/His-tag (OD 450 nm: 2,82) similar a Tropo/GST-tag (OD 450 nm: 2,40) el día 60.

**Figura 3:** Resultados de la prueba ELISA indirecta para detectar anticuerpos IgG anti-Tropo/His-tag y anti-Tropo/GST-tag en conejos inmunizados con proteínas recombinantes de tropomiosina. C1 y C2 representan conejos inmunizados con Tropo/His-tag; C3 y C4 representan conejos inmunizados con Tropo/GST-tag. El día 0 representa el suero recolectado antes de la inmunización.

El Western blot confirmó la antigenicidad de las proteínas recombinantes obteniendo una única banda con el peso esperado de 33,5 kDa para Tropo/His-tag y 60,1 kDa para Tropo/GST-tag. El antígeno recombinante reaccionó fuertemente con anti-Tropo/His-tag y anti-Tropo/GST-tag producidos en los conejos inmunizados (Figura 4). Tropo/His-tag reaccionó a una concentración de 0,001 µg, a diferencia de Tropo/GST-tag, que reaccionó a una concentración de 0,1 µg. El antígeno recombinante mostró reactividad con los anticuerpos anti-EgAPM (Figura 4).

**Figura 4:** *Western blot* de la tropomiosina recombinante reconocida por suero de conejos inmunizados. Los carriles 1 a 6 representan suero de conejos inmunizados con Tropo/His-tag y Tropo/GST-tag. Los carriles 7 a 12 representan suero de conejos inmunizados con EgAPM (antígeno nativo de *E. granulosus*). Los carriles 3, 6, 9 y 12 representan un conjunto de muestras de suero negativas de conejos antes de la inmunización. El carril M representa un marcador de peso molecular comercial.

## DISCUSIÓN

*E. granulosus* es un parásito genéticamente diverso que exhibe características, biológicas, bioquímicas, inmunológicas y antigénicas variables. Es importante considerar los tipos de proteínas de origen parasitario a utilizar, dado que las etapas larvarias y adultas producen diversos antígenos que desencadenan diferentes mecanismos de inmunidad o respuesta ^14^. En *E. granulosus*, la tropomiosina está presente en la fase larvaria y adulta y es conocida por tener una alta inmunogenicidad ^15^. En este estudio seleccionamos EgTrpA porque se ha reportado que es la forma más abundante en comparación con otras isoformas proteicas ^16^.

Se seleccionó *E. coli* como sistema de expresión debido a que es el huésped más utilizado y a menudo se lo ha descrito como eficiente. Existen sistemas más complejos que permiten la producción de proteínas con características homólogas a proteínas nativas. Sin embargo, se ha acumulado una considerable experiencia y practicidad en el cultivo de *E. coli*, lo que ha permitido mejorar significativamente la producción sin presentar complicaciones ^18^.

Los vectores plasmídicos seleccionados en este estudio se han utilizado ampliamente para producir proteínas recombinantes antigénicas ^17^. Una ventaja de utilizar vectores comerciales es la presencia de la cola de His-tag y GST-Tag que permite la purificación mediante cromatografía de afinidad. Una desventaja del vector pET28 puede ser las altas concentraciones de proteína generadas mediante este sistema que pueden dar lugar a cuerpos de inclusión ^18^. En nuestro estudio, observamos de manera similar una alta concentración de proteínas Tropo/His-tag que resultó en dificultades de purificación. El vector pGEX6P-1 produjo una baja concentración y necesitó más tiempo de producción. Mientras que una ventaja es la presencia de la proteína GST como más soluble y antigénica, lo que puede aprovecharse en el desarrollo de candidatos vacunales ^8^.

La 2-DE ha contribuido significativamente al mapeo y caracterización proteómica ^13^. En el presente estudio, antes de la purificación, se separaron de forma reproducible varios spots de proteína mediante 2-DE de alta resolución. Después de la purificación, las muestras tratadas con el kit de limpieza no dieron un mayor número de spots con la etiqueta Tropo/GST. Aun así, 2-DE permitió identificar la ausencia de cambios en la expresión de proteínas, porque, la distribución de los perfiles proteicos recombinantes fue similar a los obtenido a partir de protoescólices ^13^.

Las proteínas recombinantes Tropo/His-tag y Tropo/GST-tag fueron funcionales e inmunogénicas, ya que la respuesta inmune produjo un aumento de IgG en conejos. Estas proteínas recombinantes podrían ser usadas como fuente antigénica para la producción de anticuerpos policlonales para el diagnóstico por Copro-ELISA en perros ^19^. Además, los anticuerpos anti-EgTrpA pueden ser purificados y evaluados para el desarrollo de pruebas serológicas en hospederos intermediarios. Como resultado, la producción de proteínas recombinantes como la EgTrpA podría ser más económica, viable y sostenible que obtener proteínas de parásitos en animales infectados naturalmente.

Este estudio tiene algunas limitaciones como por ejemplo que la proteína Tropo/GST-tag recombinante no se produjo en una cantidad suficiente para inmunizar un número amplio de animales. Se requirieron varias repeticiones del protocolo para obtener suficiente proteína, por tanto, sería necesario evaluar el uso de biorreactores. Otra limitación fue que no se realizó la genotipificación del parásito, aunque se sabe que los quistes de ovino son originados exclusivamente por el genotipo G1 en Perú que afecta además comúnmente a humanos _^20^_. Finalmente, el co 3nejo como modelo animal no asemeja al huésped definitivo. Los anticuerpos no necesariamente representarían un correlato de protección, por lo que debe evaluarse la reducción de huevos en el perro.

En conclusión, este estudio describe la producción exitosa de EgTrpA en dos vectores Trpop/His-tag y Tropo/GST-tag con características homólogas a proteínas nativas del parásito. La producción de antígenos recombinantes representa una perspectiva de aplicación para el diseño y desarrollo de una vacuna contra la equinococosis en perros que requiere estudios adicionales para evaluar su eficacia. Esto sería complementario a las medidas preventivas que deben seguir articulándose bajo el enfoque Una Sola Salud, ya que en el ciclo biológico intervienen personas, cánidos silvestres y domésticos, ganado, además de la contaminación del ambiente con los huevos del parásito.
